# Slowing Development Facilitates *Arabidopsis mgt* Mutants to Accumulate Enough Magnesium for Pollen Formation and Fertility Restoration

**DOI:** 10.3389/fpls.2020.621338

**Published:** 2021-01-20

**Authors:** Xiao-Feng Xu, Xue-Xue Qian, Kai-Qi Wang, Ya-Hui Yu, Yu-Yi Guo, Xin Zhao, Bo Wang, Nai-Ying Yang, Ji-Rong Huang, Zhong-Nan Yang

**Affiliations:** Shanghai Key Laboratory of Plant Molecular Sciences, College of Life Sciences, Shanghai Normal University, Shanghai, China

**Keywords:** Mg^2+^ transporter, male fertility, Arabidopsis, MGT5, MGT6

## Abstract

Magnesium (Mg) is an abundant and important cation in cells. Plants rely on Mg transporters to take up Mg from the soil, and then Mg is transported to anthers and other organs. Here, we showed that *MGT6+/−* plants display reduced fertility, while *mgt6* plants are fertile. *MGT6* is expressed in the anther at the early stages. Pollen mitosis and intine formation are impaired in aborted pollen grains (PGs) of *MGT6+/−* plants, which is similar to the defective pollen observed in *mgt5* and *mgt9* mutants. These results suggest that Mg deficiency leads to pollen abortion in *MGT6+/−* plants. Our data showed that *mgt6* organs including buds develop significantly slower and *mgt6* stamens accumulate a higher level of Mg, compared with wild-type (WT) and *MGT6+/−* plants. These results indicate that slower bud development allows *mgt6* to accumulate sufficient amounts of Mg in the pollen, explaining why *mgt6* is fertile. Furthermore, we found that *mgt6* can restore fertility of *mgt5*, which has been reported to be male sterile due to defects in Mg transport from the tapetum to microspores and that an additional Mg supply can restore its fertility. Interestingly, *mgt5* fertility is recovered when grown under short photoperiod conditions, which is a well-known factor regulating plant fertility. Taken together, these results demonstrate that slow development is a general mechanism to restore *mgt*s fertility, which allows other redundant magnesium transporter (MGT) members to transport sufficient Mg for pollen formation.

## Introduction

Magnesium (Mg) is one of the most abundant and essential divalent metal cations in prokaryotic and eukaryotic cells. The most important function of Mg is to act as a cofactor of many enzymes. For example, Mg is essential for DNA and RNA polymerases, aminoacyl-tRNA synthetases, and ATPase. Mg-dependent enzymes participate in many metabolic and developmental processes, such as protein and nucleic acid synthesis, lipid and carbohydrate metabolism, and membrane stability (Rissler et al., 2002; Ador et al., 2004; Dudev and Lim, 2013; Gao and Yang, 2016; Li et al., 2016; Apell et al., 2017). In humans, disturbance of Mg metabolism leads to various diseases, such as heart disease, diabetes mellitus, Parkinson’s disease, Alzheimer’s disease, cancer, and growth retardation (Glasdam et al., 2016). In plants, a lack of Mg affects normal photosynthesis, growth development, and fertility (Rissler et al., 2002; Gebert et al., 2009; Xu et al., 2015).

For animals, it is easy to intake sufficient amounts of Mg by consuming Mg-containing foods such as leaves, seeds, and seafood (Glasdam et al., 2016). However, land plants obtain Mg from the soil only by their roots. Mg uptake and the subsequent transport to different organs rely on Mg transporters, which have been widely identified in both prokaryotes and eukaryotes (Snavely et al., 1989; Schmitz et al., 2003). In *Arabidopsis*, the magnesium transporter (MGT) family includes nine members (Schock et al., 2000; Li et al., 2001). *MGT1*, *MGT2*, *MGT3*, *MGT6*, and *MGT7* are expressed in the roots and are involved in Mg uptake and distribution. The *mgt1mgt2mgt3*, *mgt6*, and *mgt7* mutants display growth retardation under low-Mg conditions (Gebert et al., 2009; Lenz et al., 2013; Mao et al., 2014; Oda et al., 2016). *MGT10* is expressed in the leaves and is essential for chloroplast development and photosynthesis (Sun et al., 2017).

In flowering plants, the stamen is a distal organ located far from the root. Pollen develops in the anther, and mature pollen is released after anther dehiscence (Xu et al., 2019). The anther consists of four microsporangiums, which are connected with vascular bundle of filament by the connective cells (Scott et al., 2004). Each microsporangium is surrounded by a layer of tapetum which provides various nutrients and materials such as sporopollenin for pollen development (Ariizumi and Toriyama, 2011; Wang et al., 2018). Mg is transported through filaments and connective cells to the four-lobed structure of anthers. In each locule, the tapetum secretes Mg to support pollen formation (Xu et al., 2015). Of the MGT family, *MGT4*, *MGT5*, and *MGT9* have been reported to be involved in pollen formation and plant fertility. Pollen of *mgt4*, *mgt5*, and *mgt9* show similar defects in intine formation and pollen mitosis, suggesting insufficient amounts of Mg in the pollen of these plants (Li et al., 2015; Xu et al., 2015). Both *MGT4* and *MGT9* are highly expressed in pollen. MGT4 is located in the endoplasmic reticulum and expressed in pollen from the bicellular to mature pollen stage (Li et al., 2015). MGT9 is located on the plasma membrane of microspores (MSps) and is involved in the absorption of Mg from locules (Chen et al., 2009; Xu et al., 2015). *MGT5* is expressed in the tapetum and MSps. It has dual localization, occurring in both mitochondria and the plasma membrane. In the tapetum, MGT5 is located in the plasma membrane and is important for Mg transport from the tapetum to the locule. Moreover, MGT5 is also essential for mitochondrial Mg homeostasis in pollen (Li et al., 2008; Xu et al., 2015). *mgt5* plants are male sterile. Under high-Mg conditions, the fertility of these plants is fully restored, suggesting that other MGTs are also involved in Mg transport (Xu et al., 2015). *MGT6* is expressed in the root and flower (Gebert et al., 2009) and is localized in the plasma membrane (Mao et al., 2014). *MGT6* is important for Mg uptake and transport, as its reduced expression in roots results in growth retardation (Mao et al., 2014; Yan et al., 2018). However, its function in anthers is unclear.

Male sterility is an important trait in agriculture. Thermosensitive genic male sterility (TGMS) and photoperiod-sensitive genic male sterility (PGMS) have been widely used in the two-line system hybrid rice breeding. In rice, several P/TGMS genes have been cloned (Chen and Liu, 2014). Our group has reported a general mechanism of fertility restoration in *Arabidopsis* (Zhu et al., 2020). Recently, the TGMS line *reversible male sterile* (*rvms*), which encodes a GDSL lipase, was cloned (Zhu et al., 2020). Additionally, several pollen wall-related *Arabidopsis* male-sterile lines were identified as TGMS lines. Their fertility was restored under low-temperature conditions. Analysis of the underlying mechanisms revealed that slow development is a general mechanism for the fertility restoration of these TGMS lines (Zhu et al., 2020). The fertility of these lines can also be restored under short days or low-light conditions. Therefore, slow development was proposed to be a general mechanism for fertility restoration of PGMS lines (Zhang et al., 2020). In this study, the partial male sterility of a heterozygous mutant of *MGT6* (*MGT6+/−*) and a fertile homozygous line (*mgt6*) were further analyzed. We dissected the complex relationship among environmental factors (light and temperature), Mg transport in the anther, plant growth and plant fertility. The results show that MGT6 plays redundant roles with other MGT members in the transport of Mg for anther development and pollen formation. Slow development is also a general mechanism to restore the fertility of *mgt* male-sterile lines.

## Materials and Methods

### Plant Material and Growth Conditions

*Arabidopsis* ecotype Columbia-0 was the source of both the WT and mutant plants. T-DNA insertion mutants of *mgt5* (SALK_037061C) and *mgt6* (Salk_203866C) were obtained from the ABRC.^1^ The *dyt1*, *tdf1*, *ams*, *myb80*, and *tek* mutants were preserved at the laboratory of Z.N.Y. The genotypes were confirmed by PCR. The plants were grown under long photoperiod (16 h of light/8 h of darkness) or short days (8 h of light/16 h of darkness) at approximately 22°C and 80% relative humidity. The soil was vermiculite and was fertilized with 0.1% chemical fertilizer (consisting of 100 μM Mg; Shanghai Yongtong Chemical Co., Ltd., China).

### Hydroponic Cultivation

The hydroponic cultivation of *Arabidopsis* was performed as described previously (Xu et al., 2015). The seeds were placed on the surface of sponges soaked with plant nitrate solution (PNS). The sponges were placed in a large opaque container that contained 4 L of PNS and that was covered with a transparent lid. The container was then transferred to a growth chamber (AR-41 L2, Percival, China). The light intensity was 100 μmol photons m^−2^ s^−1^. The bottom of the sponge was submerged in the PNS to prevent desiccation. After 10 days, healthy seedlings were retained from each sponge. After approximately 20 days, the seedlings were transferred to a larger opaque container. This container contained 5 L of PNS with various concentrations of Mg.

### Microscopy

The plants were imaged with a Cybershot T-20 digital camera (Sony, Japan). Silique images were acquired under a dissecting microscope equipped with a DP70 digital camera (Olympus, Japan). Alexander staining solution (Alexander, 1969) was prepared containing 10 ml of ethanol, 1 ml of 1% malachite green in 95% ethanol, 50 ml of distilled H_2_O, 25 ml of glycerol, 5 g of phenol, 5 g of chloral hydrate, 5 ml of 1% acid fuchsine in H_2_O, 0.5 ml of 1% orange G in H_2_O, and 1–4 ml of glacial acetic acid. The anthers were stained overnight at room temperature. The flower buds for semithin sections were prepared and embedded in Spurr’s resin. The semithin sections were prepared at a thickness of 1 μm and incubated in a 0.01% toluidine blue/sodium borate solution for 5 min at 45°C before being washed with H_2_O. The sections were subsequently observed under bright-field microscopy.

### Electron Microscopy

For SEM, fresh pollen grains (PGs) were coated with 8 nm of gold and observed under a JSM-840 microscope (JEOL, Japan). For TEM, the flower buds were fixed in 0.1 M phosphate buffer (pH 7.2) comprising 2.5% glutaraldehyde (v/v) and then washed several times before gradient dehydration. Finally, the samples were embedded in Spurr’s resin and polymerized for 48 h at 60°C. The resulting ultrathin sections were examined *via* transmission electron microscopy (JEOL, Japan).

### Plasmid Construction and Identification of Transgenic Plants

The *MGT6* genomic region was cloned using primers (CMGT6-F/R) to complement the *mgt6* and *MGT6+/−* mutants. The fragment was amplified using KOD polymerase (Takara Biotechnology, http://www.takarabio.com) and cloned into a pCAMBIA1300 binary vector (Cambia, Australia). The CRISPR/Cas9 system was used for gene editing of *MGT6*. MGT6-Cas9-F/R primers were used to generate *MGT6-gRNA* in the backbone vector: pEarlyGate100 which was modified with *Arabidopsis* YAO promoter-driven Cas9 (Yan et al., 2015). The plasmids were transformed into *Agrobacterium tumefaciens* GV3101 and screened using 50 mg/ml kanamycin, 40 mg/ml gentamicin, and 50 mg/ml rifampicin. The agrobacteria containing the plasmid constructs were introduced into the plants. Transformed *mgt6* and *MGT6+/−* lines carrying the *MGT6* complement fragment were selected using 20 mg/L hygromycin. PCR was performed to verify the backgrounds of the transgenic lines. The primers MGT6ID-F/R, MGT6IDF/CMGT6IDR, and SalkLB1.3/MGT6IDR were used to identify the genotype of the T-DNA insertion. The primer MGT6IDF/1300P2 was used to confirm the insertion of the complement fragment of the *MGT6* genome. Transformed WT plants carrying the pYAOCRISPR/Cas9-MGT6-gRNA construct was selected using 0.1% Basta. The editing sites in the transgenic lines were verified by sequencing of the gene specific PCR product. The sequences of the primers used are listed in Supplementary Table S2.

### RT-PCR and qRT-PCR

RNA was extracted from the inflorescences of the wild-type (WT) and mutant plants using a TRIzol kit (Invitrogen, United States). Reverse transcription PCR (RT-PCR) was performed for 28 cycles to analyze the expression of *MGT6*, using the primers rtMGT6-F/R. Quantitative reverse transcription PCR (qRT-PCR) was performed on an ABI PRISM 7300 detection system (Applied Biosystems, USA) in conjunction with SYBR Green I master mix (Toyobo, Japan). The expression level of *MGT6* was detected using primers qMGT6-F/R. The qRT-PCR results were shown as the relative expression levels normalized to those of *TUBULIN BETA 8* (*Tubulin*), which served as the positive control (Tub-F/R). The primers used for RT-PCR and qRT-PCR are listed in Supplementary Table S2.

### RNA *in situ* Hybridization

For RNA *in situ* hybridization, the protocol was performed as described previously (Xu et al., 2015). Here, the flower samples were fixed in FAA solution (RNase free), dehydrated in an ethanol gradient, and embedded in wax. The sections were prepared using a rotary microtome (MR2; RMC, United States). For RNA *in situ* hybridization, a 108 bp *MGT6* probe (inMGT6-F/R) was synthesized with a DIG RNA Labeling Kit (Roche, United States). The sections were incubated with xylene and rehydrated in an ethanol series. Proteinase K (Roche)-treated slides were hybridized with the probe at 55°C overnight. The slides were blocked with 1% blocking reagent (Roche) in a solution consisting of 100 mM Tris (pH 7.5) and 150 mM NaCl. An anti-DIG antibody (Roche) was used for immunological detection, and an NBT/BCIP solution (Roche) was diluted 1:100 in a solution consisting of 100 mM Tris (pH 9.5), 100 mM NaCl, and 50 mM MgCl_2_ for colorimetric detection at room temperature. The sections were ultimately imaged under an Olympus BX51 microscope.

### GUS Analysis

The construction of *Arabidopsis* lines carrying *promoterMGT6::GUS* fusion construct is described in a previously published report (Gebert et al., 2009). Here, flowers were collected and incubated in β-glucuronidase (GUS) staining solution at 37°C for 12 h. The samples were then decolorized in a gradient of ethanol solutions (30, 50, 60, and 70%) or further dehydrated in an ethanol gradient (80, 90, 95, and 100%), after which they were embedded in wax before sectioning. The flower samples were imaged under an Olympus SZX12 microscope. The sections of anther samples were imaged under an Olympus BX51 microscope.

### Measurement of Mg Concentration

The Mg^2+^ concentrations in the stamens of *Arabidopsis* WT, *MGT6+/−* and *mgt6* plants grown in the same pot containing approximately 100 μM Mg^2+^ were measured. After 6 weeks, the fresh stamens during anther stages 5–9 were collected and weighed (>10 mg). During stage 5–9, the bude diameters ranged from 400 to 650 μm. The anther stages were determined by semi-thin section (Sanders et al., 1999). The stamen samples were first digested with HNO_3_ and maintained at 98°C for 3 h, and then ultrapure H_2_O of the same volume was added. The concentration of Mg^2+^ was measured *via* inductively coupled plasma optical emission spectrometry (ICP-OES; Vista-MPX, America) at the 285.2 nm wavelength. The Mg^2+^ content in ultrapure H_2_O used in this assay was 0.005814 mg/L.

## Results

### Phenotypes of Fertile *mgt6* and Partial Male Sterile *MGT6+/−*

**Figure 1:**
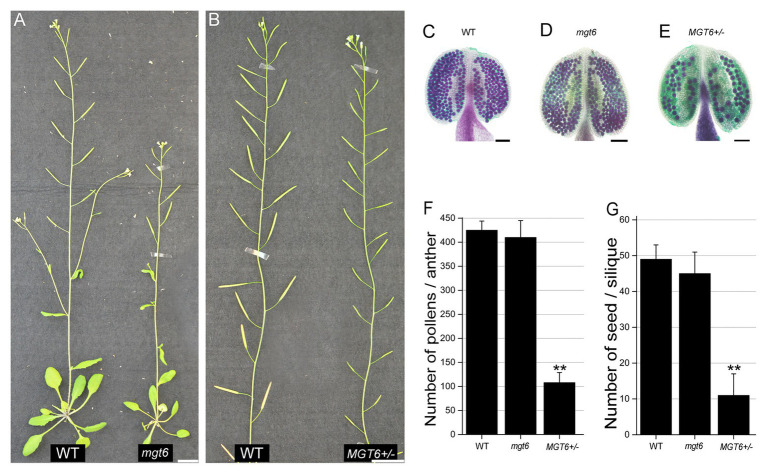
Heterozygous *MGT6+/−* mutants exhibit severe sterility. **(A)** Phenotypes of the wild-type (WT) and homozygous mutant of *MGT6* (*mgt6*). **(B)** Phenotypes of the WT and heterozygous mutant of *MGT6* (*MGT6+/−*). Bars, 1.5 cm. **(C–E)** Alexander staining of anthers of WT **(C)**, *mgt6* **(D)**, and *MGT6+/−* **(D)**. The mature pollen is purple, but the aborted pollen is green. Bars, 1 mm. **(F,G)** Number of pollens **(F)** and seeds **(G)** in each anther and silique from WT, *mgt6*, and *MGT6+/−* plants. The means are shown with ±SDs, *n* > 30. A two-sample *t*-test was used to evaluate statistical significance compared with the WT (^**^ *p* < 0.01).

To investigate the role of *MGT6* in plant reproduction, a T-DNA insertion line, Salk_203866C, was obtained from the Arabidopsis Biological Resource Center (ABRC). PCR-based analysis and sequencing confirmed that the T-DNA insertion was present in the third exon of the *MGT6* gene in this line (Supplementary Figures S1A,B). The leaves of *mgt6* are smaller than those of the wild type (WT) under low-Mg conditions, though this phenotype was rescued under high-Mg conditions (Supplementary Figure S2). This is consistent with the findings of previous studies (Mao et al., 2014; Oda et al., 2016; Yan et al., 2018). *mgt6* displayed a dwarf phenotype and the same fertility as WT (Figure 1A). Then we obtained heterozygous T-DNA insertion plants (*MGT6+/−*) from cross between *mgt6* and WT. In contrast, the *MGT6+/−* showed no observable defects in vegetative growth, but abnormal fertility, as indicated by its relatively short siliques (Figure 1B; Supplementary Figure S3). Alexander staining revealed that, like that of the WT, the pollen fertility of *mgt6* was normal. However, the majority of PGs were aborted in *MGT6+/−* anthers (Figures 1C–E). To further confirm the phenotype of *MGT6+/−*, we generated another heterozygous mutant of *MGT6* by the use of the CRISPR/Cas9 system (*cas9MGT6+/−*). Like *MGT6+/−*, the *cas9MGT6+/−* mutant exhibited severe sterility and reduced pollen fertility (Supplementary Figure S3). Statistical analysis showed that there were approximately 400 pollen grains in each WT anther. In each *mgt6* anther, the number of pollen grains was similar to that in the WT anther. However, the amount of pollen grains in the anthers from *MGT6+/−* was only approximately 25% of that in the WT anthers (Figure 1F). Furthermore, the number of seeds was severely decreased in *MGT6+/−* plants, compared with the WT and *mgt6* plants (Figure 1G). These suggest that *MGT6+/−* affects normal pollen development and plant fertility.

To complement the *mgt6* and *MGT6+/−* mutant phenotype, the 3.1 kb genomic fragment of the *MGT6* gene was cloned from the WT and transferred to *mgt6* and *MGT6+/−* plants. In the T_2_ generation, 22/22 transgenic lines in the *mgt6* background showed normal vegetative growth and fertility. Moreover, 5/5 transgenic lines in the *MGT6+/−* background showed normal fertility (Supplementary Figures S1C–E). These results demonstrated that the T-DNA insertion in *MGT6* affects the normal function of this gene during plant reproduction. RT-PCR indicated that the expression of *MGT6* was completely absent in the *mgt6* mutant. qRT-PCR showed that the amount of *MGT6* transcripts was half as low in the *MGT6+/−* flowers than in the WT flowers. Furthermore, the expression of *MGT6* in the inflorescences was not affected when the plants were grown under different Mg conditions (Supplementary Figure S4). These results suggest that *MGT6* is involved in male fertility.

### *MGT6* Functions in the Sporophytic Tissue of Anthers for Pollen Formation

**Figure 2:**
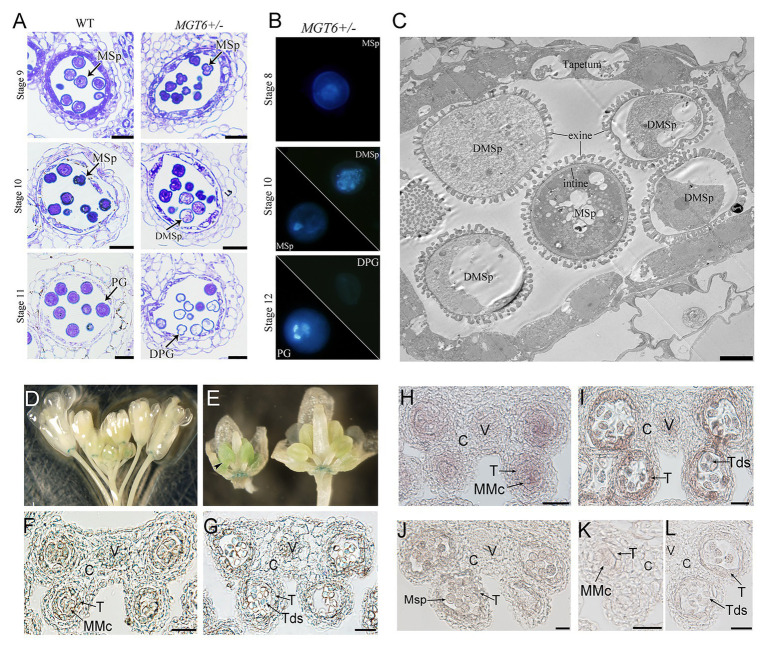
Pollen development defects in *MGT6+/−* mutants and *MGT6* expression patterns in anthers. **(A)** Semithin sections of anthers of WT and *MGT6+/−* mutants showing pollen development at the anther stages 9–11. Bars, 20 μm. The anther developmental stages refer to previous report (Sanders et al., 1999). **(B)** DAPI staining indicates male gametophytic mitosis within normal and aborted pollen of *MGT6+/−* plants. **(C)** TEM of anthers of *MGT6+/−* plants. The image shows that the intine development of aborted microspores (MSps) is prevented in the *MGT6+/−* mutant. Bar, 1 μm. DPG, degenerated pollen grain; DMSp, degenerated microspore; MSp, microspore; and PG, pollen grain. **(D–G)** GUS expression in inflorescences of transgenic plants harboring the *promoterMGT6::GUS* construct. The black arrow indicates the expression of GUS in the anthers of young buds **(D,E)**. Paraffin sections of GUS-stained anthers. GUS expression in transverse sections of anthers transformed with *promoterMGT6::GUS* at stage 5 **(F)** and stage 7 **(G)**. **(H–L)** RNA *in situ* hybridization in which a *MGT6* antisense probe was used to detect *MGT6* expression in WT anthers at stages 6–8. **(K,L)** A *MGT6* sense probe was used as a control at stage 5 **(K)** and 7 **(L)**. At least 30 anthers were examined in each stage under the microscope. C, connective cell; V, vascular region; T, tapetum; Tds, tetrads; and MMc, microspore mother cell. Bars, 20 μm.

Cytological analyses were employed to understand the defects of anther development in the *MGT6+/−* plants. Semithin sections revealed that anther development is normal in the *MGT6+/−* mutant. No significant difference was observed during anther stages 4–8 (Sanders et al., 1999) between *MGT6+/−* and WT. At later stages, no significant developmental defects were found in the vascular, connective, or tapetal cells. However, most *MGT6+/−* microspores began degenerating at stage 10 and had aborted at stage 11 (Figure 2A). DAPI staining indicated that meiosis is normal in *MGT6+/−* plants. However, some of the microspores displayed diffuse staining in *MGT6+/−* cells at stage 10. At stage 12, normal pollen with two sperm cells and one vegetative cell was formed, but no signal was observed in the aborted pollen in *MGT6+/−* (Figure 2B). Ultrathin sections revealed that the intine was absent in aborted pollen, while the exine was not affected in the *MGT6+/−* pollen (Figure 2C). These results show that normal mitosis and intine development are defective in aborted pollen from the *MGT6+/−* mutant. The developmental defects in the aborted pollen from *MGT6+/−* are similar to those in pollen from *mgt4*, *mgt5*, and *mgt9* (Li et al., 2015; Xu et al., 2015). Thus, it is possible that insufficient amounts of Mg lead to pollen abortion in *MGT6+/−* plants.

During anther development, the tapetum secretes Mg into the locules, where microspores take it up for pollen formation. It has been shown that *MGT4* and *MGT9* mainly function in pollen (Chen et al., 2009; Li et al., 2015), whereas MGT5 functions in both pollen and the tapetum (Xu et al., 2015). To understand whether *MGT6* functions in sporophytes and/or gametophytes, a transmissibility assay was performed. WT plants were crossed with *MGT6+/−* plants. The reciprocal cross experiments revealed that the transmission of *MGT6+/+* and *MGT6+/−* alleles is close to 1:1 in the F_1_ generation, suggesting that gametophytes with T-DNA insertion have normal fertility (Supplementary Table S1). Furthermore, the *MGT6+/+* (normal), *MGT6+/−* (partial male sterile), and *mgt6* (dwarf) plants of population from *MGT6+/−* segregated with the ratio of 1:2:1 (*n* > 200). These results indicate that MGT6 functions in sporophytic tissue to supply Mg for microspore development. To address the expression pattern of *MGT6* in flowers, we obtained the *promoterMGT6::GUS* transgenic plants that had been previously generated (Gebert et al., 2009). GUS signals were observed in the anthers of young buds (Figures 2D,E) and were consistently expressed in the receptacle (Supplementary Figure S5). Paraffin sections showed that GUS signals occurred in the vascular region and connective, tapetal and male gametophytic cells during anther stages 5–7 (Figures 2F,G). Furthermore, RNA *in situ* hybridization assays showed that *MGT6* was expressed in male gametophytic cells and surrounding sporophytic cells during anther stages 5–7 (Figures 2H–L), which is consistent with the GUS staining. These results suggest that *MGT6* mainly functions in Mg transport in sporophytic tissues of anthers during the early stage. In anther, the tapetum is one of the important sporophytic cells for pollen development. There are five transcription factors, DYT1, TDF1, AMS, MYB80, and TEK are essential for tapetum development and function (Lou et al., 2014). The *MGT5* is under the control of DYT1-TDF1-AMS pathway in tapetum (Xu et al., 2015). To understand whether this genetic pathway also regulates *MGT6*, we analyzed the expression level of *MGT6* in these mutants. The qRT-PCR indicated that the expression of *MGT6* was not affected in these mutant inflorescences (Supplementary Figure S5). This suggest that the *MGT6* may be independent of this pathway in the tapetum.

### The Slow Growth of *mgt6* Buds Results in the Accumulation of Excess Mg

**Figure 3:**
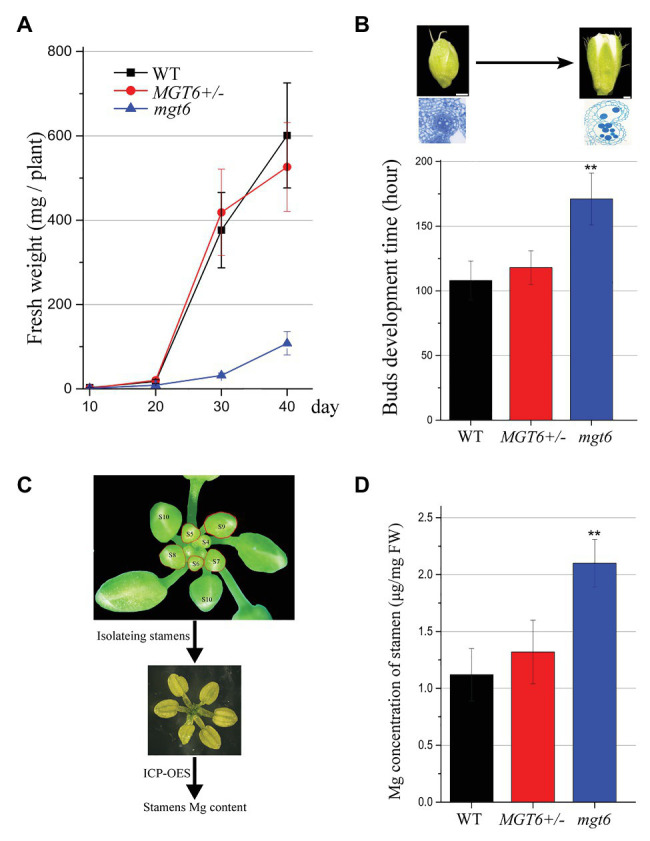
Flower bud development time and the magnesium (Mg) concentration in the stamen. **(A)** Fresh weight of WT, *MGT6+/−*, and *mgt6* plants. The plants were grown under long photoperiod and supplied with 100 μM Mg. The error bars indicate SDs and were calculated from three replicates. **(B)** Quantitative analyses of floral bud development time for WT, *MGT6+/−*, and *mgt6* plants under long photoperiod. Measurement of the development time of buds during anther stages 5 to 13. The means are shown as ±SDs of three biological repeats, *n* > 30. Bars, 200 μm. A two-sample *t*-test was used to evaluate statistical significance compared with the *MGT6+/−* (^**^ *p* < 0.01). **(C,D)** Mg concentrations of stamens in the flower buds of the WT, *MGT6+/−*, and *mgt6* plants. Fresh stamens at anther stages 5–9 were isolated from buds **(C)**. The stamens were weighed to determine the Mg concentration *via* inductively coupled plasma optical emission spectrometry (ICP-OES; **D)**. The means are shown with ±SEs of three technical repeats. A two-sample *t*-test was used to evaluate statistical significance compared with the *MGT6+/−* (^**^ *p* < 0.01).

Our previous results have shown that slow development restores the fertility of P/TGMS lines (Zhang et al., 2020; Zhu et al., 2020). The absence of *MGT6* function can result in developmental retardation (Mao et al., 2014). We analyzed the overall developmental status of *mgt6* and *MGT6+/−* mutants under the same growth conditions. The measurement of fresh weight indicated that the growth speed of *mgt6* was much slower than that of WT and *MGT6+/−* (Figure 3A). The *MGT6+/−* fertility was dramatically reduced compared with that of WT and *mgt6* (Figure 1). Previous investigations have shown that low-temperature treatment is effective at the reproductive stage (Zhu et al., 2020). We further analyzed the developmental speed of the flower buds of *MGT6+/−* and *mgt6*. The young floral buds were marked, and the time needed to grow from stage 5 to 13 was recorded. Quantitative analysis showed that the flower bud development of *mgt6* was prolonged compared with that of WT and *MGT6+/−* (Figure 3B), indicating that the flower bud development of *mgt6* is slower than that of WT and *MGT6+/−*. Taken together, these results suggest that slow development may contribute to fertility restoration in *mgt6*.

Magnesium is essential for microspore development and pollen formation (Xu et al., 2015). We further analyzed whether *mgt6* plants accumulated sufficient amounts of Mg for pollen formation. *MGT6* is expressed in anther tissues at early stages (Figure 2). We isolated fresh stamens from young buds (anther stages 5–9) of WT, *MGT6+/−*, and *mgt6*. ICP-OES was employed to measure the Mg concentrations in the fresh stamens (Figure 3C). The ICP-OES analysis results showed that the *MGT6+/−* stamens contain Mg in amounts similar to those in the WT stamens. However, the *mgt6* stamens contained more Mg than the WT and *MGT6+/−* stamens (Figure 3D). Therefore, these results suggest that slow development facilitates Mg accumulation in pollen, which leads to fertility restoration in *mgt6*.

### *mgt6* Restores the Fertility of *mgt5*

**Figure 4:**
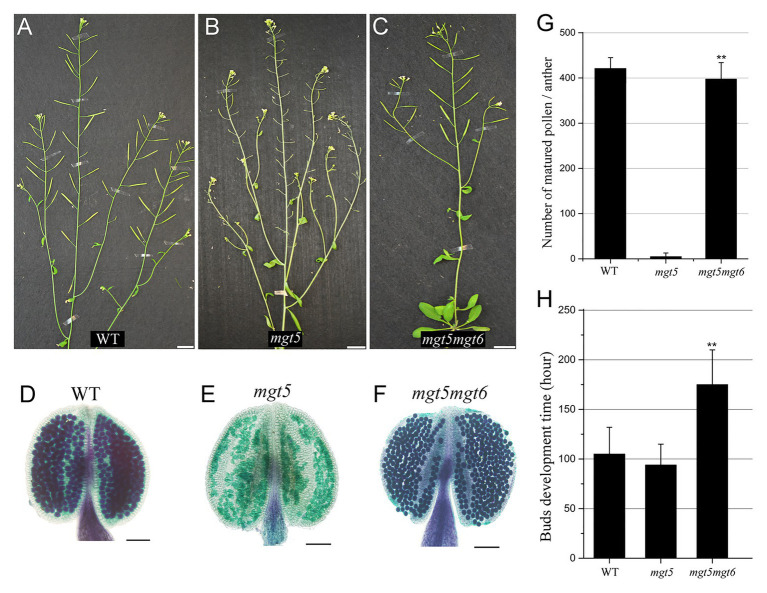
Phenotypes of *mgt5* and *mgt5mgt6* under long-photoperiod conditions. **(A–C)** Phenotypes of WT **(A)**, *mgt5* **(B)**, and *mgt5mgt6* **(C)** under long-day conditions. The short siliques indicate that *mgt5* is sterile, while *mgt5mgt6* shows normal fertility. Bars, 1.5 cm. **(D–F)** Alexander staining of the anthers from WT **(D)**, *mgt5* **(E)**, and *mgt5mgt6* **(F)** plants grown under long-photoperiod conditions. Bars, 1 mm. **(G)** The numbers of pollen in each anther from WT, *mgt5*, and *mgt5mgt6*. The means are shown with ±SDs, *n* > 30. **(H)** Quantitative analyses of flower bud development time for WT, *mgt5*, and *mgt5mgt6* plants under long-photoperiod conditions. The means are shown as ±SDs of three biological repeats, *n* > 30. A two-sample *t*-test was used to evaluate statistical significance compared with the *mgt5* (^**^ *p* < 0.01).

Slow development under low temperature and/or short photoperiod restores the fertility of *Arabidopsis* P/TGMS lines (Zhang et al., 2020; Zhu et al., 2020). Compared with the WT, the *mgt6* mutant is slower in buds development and contains high Mg accumulation in anther (Figure 3). We crossed *mgt6* with *mgt5* to obtained *mgt5mgt6* double mutants. *mgt5* plants show severe male sterility under low Mg condition (Xu et al., 2015). Like *mgt6*, the *mgt5mgt6* mutant showed overall developmental retardation but normal fertility (Figures 4A–C). Pollen development was restored in the *mgt5mgt6* mutant compared with the *mgt5* mutant under the same developmental conditions (Figures 4D–G), indicating that pollen development of *mgt5* was restored by *mgt6*. The bud development of the *mgt5mgt6* plants was slower than that of the WT and *mgt5* plants (Figure 4H). Previous work has shown that MGT5 is important for Mg transport from the tapetum to pollen. The *mgt5* pollen can restore fertility under high Mg conditions (Xu et al., 2015). These findings indicate that slow development in *mgt6* also facilitates Mg accumulation in *mgt5* pollen.

### Short Photoperiod Restores *mgt5* Fertility

**Figure 5:**
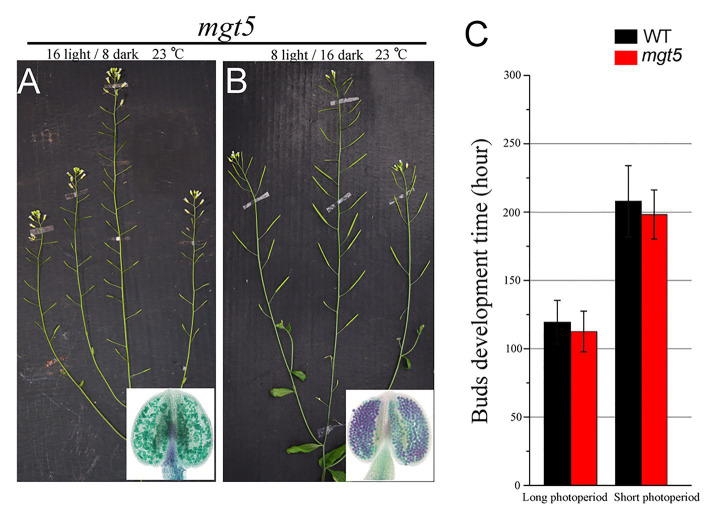
Phenotypes of *mgt5* under different photoperiod conditions. **(A,B)** Fertility of *mgt5* under short-photoperiod and long-photoperiod conditions in the presence of 100 μM Mg. Alexander staining of the anthers from these plants. **(C)** Quantitative analyses of flower bud development time for WT and *mgt5* under long-photoperiod and short-photoperiod conditions. The means are shown as ±SDs of three biological repeats, *n* > 30.

The *mgt5* mutant shows severe male sterility, but its fertility was restored under high-Mg conditions (Xu et al., 2015). Fertility restoration of P/TGMS lines under low temperature and/or short photoperiod has been widely applied in two-line rice breeding systems (Chen and Liu, 2014). We analyzed whether these environmental factors also affect the fertility of *mgt5*. To this end, we grew WT and *mgt5* plants under different photoperiod and temperature conditions as reported previously (Zhang et al., 2020; Zhu et al., 2020). The buds development speed of these plants under short-photoperiod or low-temperature conditions was greatly reduced. Interestingly, we found that *mgt5* showed normal fertility under short-photoperiod conditions, but not under low-temperature conditions. Alexander staining indicated that the *mgt5* pollen formed under short-photoperiod conditions but not under low-temperature conditions (Figures 5A,B; Supplementary Figure S6). The growth speeds of WT and *mgt5* plants were greatly reduced under short-photoperiod conditions, and the flower bud development time was greatly prolonged (Figure 5C). These results suggest that a short photoperiod leading to slow bud development can satisfy Mg requirement for pollen development.

## Discussion

### MGT6 Plays Redundant Roles With Other MGT Members in the Transport of Mg for Anther Development and Pollen Formation

**Figure 6:**
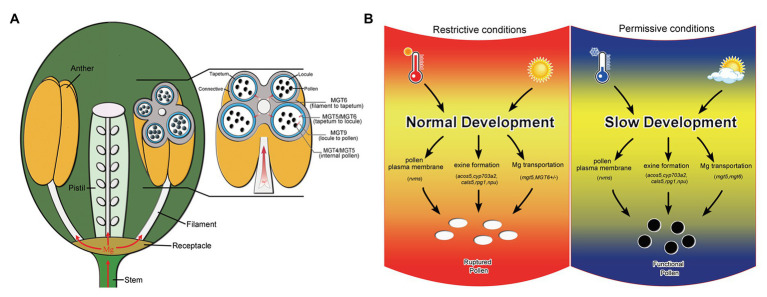
Model of Mg transport in anthers and schematic diagram of the mechanism by which slow development restores fertility. **(A)** Model of the Mg transport pathway in anthers. *MGT6* is responsible for Mg transport from the filaments to the tapetum. *MGT5* and *MGT6* are involved in Mg transport from the tapetum to the locules. *MGT9* is essential for pollen absorption of Mg from the locules. Both *MGT5* and *MGT4* are important for Mg homeostasis in pollen. **(B)** Schematic diagram of the mechanism by which slow development functions in fertility restoration, modified from our previous report (Zhang et al., 2020). The *rvms*, *acos5-2*, *cyp703a2*, *cals5*, *rpg1*, and *npu* show male sterility and defective pollen development. Temperature and photoperiod are important for the fertility restoration of these T/photoperiod-sensitive genic male sterility (PGMS) lines (Zhang et al., 2020; Zhu et al., 2020). Under restrictive conditions, pollen development is also affected in the *mgt5* and *MGT6+/−* mutants (left). However, Mg accumulation can be restored by slow development in *mgt6* and under short-photoperiod conditions in *mgt5* (right).

Magnesium is essential for vegetative growth and male reproduction (Gebert et al., 2009; Mao et al., 2014; Xu et al., 2015). Mg uptake and transport mainly rely on members of the MGT family in *Arabidopsis* (Gebert et al., 2009). Several MGT members have been reported to be involved in pollen formation. In this study, we reveal that *MGT6* is also involved in Mg transport in the anthers for pollen formation (Figure 1). The defects in pollen intine and mitosis in *MGT6+/−* (Figure 2) are quite similar to those in *mgt5* and *mgt9* (Xu et al., 2015). This suggests that the amount of Mg is not sufficient to support pollen formation in *MGT6+/−*. Many MGT members are expressed in the anthers (Gebert et al., 2009), which are extremely complex reproductive organs in plants (Xu et al., 2019). The sporophytic tissues in anthers must transport sufficient amounts of Mg for pollen formation during plant reproduction. The transport of nutrients to pollen depends on the coordination of different types of sporophytic cells (Scott et al., 2004). *MGT5* is expressed in the tapetum and microspores during anther stages 6–7 (Xu et al., 2015). *MGT9* expression clearly occurs in the tapetum and microspores during anther stages 7–9 (Chen et al., 2009). Therefore, these two genes play important roles in Mg transport from the tapetum to microspores during anther stages 6–9. Genetic analysis indicated that MGT6 plays a sporophytic role in pollen formation (Supplementary Table S1). In the anthers, *MGT6* is expressed in the vascular region, connective cells, tapetum, and microspores during stages 5–7 (Figure 2). These results suggest that MGT6 mainly functions in Mg transport from the filament to the tapetum. We thus propose a model to summarize Mg transport in the anthers: MGT6 is responsible for Mg transport from the filaments to the tapetum; MGT5 is involved in Mg transport from the tapetum to the locule; MGT9 is essential for microspores to obtain Mg from the locules; and finally, MGT4 and MGT5 maintain Mg homeostasis in the pollen (Figure 6A). All MGT family members function in Mg transport, and protein-protein interactions between different members are highly permissive (Schmitz et al., 2013). Both MGT5 and MGT6 are localized in the plasma membrane (Mao et al., 2014; Xu et al., 2015), and they are expressed in tapetum at stages 6–7 (Figure 2; Xu et al., 2015). Hence, MGT6 may interact with MGT5 to facilitate Mg transportation from tapetum to locule so that pollen/microspore can obtain sufficient Mg under different conditions. The *mgt6* showed normal pollen fertility, and its stamen contains higher Mg than WT and *MGT6+/−* (Figures 1, 3), suggesting that higher Mg in stamens can overcome the Mg transportation defects in *mgt6* anther. The fertility restoration of *mgt5* and *mgt6* under different conditions suggests that other MGT members are also involved in Mg transport in the stamen.

### Slow Development Allows *mgt* Mutants to Accumulate Sufficient Amounts of Mg for Pollen Formation

Male sterility and fertility restoration are important not only for understanding plant development and reproduction but also for plant breeding with respect to agriculture (Chen and Liu, 2014). The formation of fine exine pattern plays an important role in determining male fertility (Guan et al., 2008; Chang et al., 2012; Huang et al., 2013; Xiong et al., 2016; Peng et al., 2020; Xue et al., 2020). A recent investigation revealed that *rvms* and other exine-defect *Arabidopsis* mutants are TGMS lines, as their fertility could be restored under low temperature; mechanism analysis revealed that slow development under low temperature restores the fertility of these TGMS lines (Zhu et al., 2020). Plants grow slowly under short photoperiod or low light; these conditions also restored the fertility of these TGMS lines (Zhang et al., 2020). Therefore, slow development is a general mechanism for fertility restoration of P/TGMS lines under low temperature and/or short photoperiod. In this work, the slow growth of *mgt6* led to fertility, while *MGT6+/−* remained male sterile (Figure 1), and *mgt6* restored the fertility of *mgt5* (Figure 4). These results demonstrate that slow development is also a general mechanism to restore the fertility of *mgt* male-sterile lines (Figure 6B). Both low temperature and/or short photoperiod slow plant development and restore P/TGMS fertility in *Arabidopsis* (Zhang et al., 2020; Zhu et al., 2020). However, the fertility of *mgt5* could be restored only under short photoperiod (Figure 5). We found that low-temperature conditions were unable to restore the fertility of *mgt5* plants (Supplementary Figure S6). It has been reported that Mg uptake and transport are sensitive to low temperature (Tanoi et al., 2014). It is likely that Mg accumulation under slow growth of low temperature is not sufficient to support microspore development or pollen formation in *mgt5* plants. *MGT6+/−* cannot restore its fertility under short-photoperiod. We observed that the buds development of *MGT6+/−* under short-photoperiod was not prolonged (Figure 3; Supplementary Figure S7). This further supports that slow development facilitates Mg accumulation in pollen. Under high Mg conditions, the *MGT6+/−* plants showed normal growth, but its fertility also remained partial sterile (Supplementary Figure S8). It was reported that MGT6 controls plant Mg homeostasis in both root and shoot tissues (Yan et al., 2018). These suggest that the *MGT6+/−* plant may not obtain extra Mg under high Mg conditions. In *rvms*, *cals5*, and other P/TGMS lines, slow development overcomes cellular defects and produces functional pollen (Zhang et al., 2020; Zhu et al., 2020). Members of the MGT family play a redundant role in Mg transport in plant development. Several other MGT family members have been reported to be expressed in the anthers (Gebert et al., 2009). The *mgt6* and *mgt5mgt6* plants under normal growth conditions and *mgt5* plants under short photoperiod grew slowly and were fertile (Figures 4, 5). Slow development may allow other redundant MGT members to transport sufficient Mg for pollen formation.

## Data Availability Statement

The original contributions presented in the study are included in the article/Supplementary Material, further inquiries can be directed to the corresponding author.

## Author Contributions

Z-NY led the project. Z-NY and X-FX designed the experiments. X-FX, X-XQ, K-QW, Y-HY, Y-YG, BW, XZ, and N-YY performed the experiments. Z-NY, J-RH, and X-FX wrote the manuscript. All authors contributed to the article and approved the submitted version.

### Conflict of Interest

The authors declare that the research was conducted in the absence of any commercial or financial relationships that could be construed as a potential conflict of interest.

## Footnotes

^1^https://abrc.osu.edu
