# Supplementary material for: Slowing Development Facilitates Arabidopsis mgt Mutants to Accumulate Enough Magnesium for Pollen Formation and Fertility Restoration
Source: Front Plant Sci. 2021 Jan 20;11:621338. doi: 10.3389/fpls.2020.621338 (PMC7854698; doi:10.3389/fpls.2020.621338)
Supplement: Supplementary file 1 [file Data_Sheet_1.pdf]

Table S1.

Transmission efficiency of the heterozygous mutant of *MGT6*.

Parental Genotypes		Genotypes of Progeny		Total	TEf	TEm
Male	Female	*MGT6+/+*	*MGT6+/-*			
WT	*MGT6+/-*	112	115	227	50.66%	NA
MGT6+/-	WT	141	135	276	NA	48.91%

The transmission efficiencies (TE) were calculated according to the following equation: TE = number of progenies with T-DNA insertion/number of total progeny × 100%. For normal transmission, 50% reciprocal crosses were expected. TEf, female transmission efficiency; TEm, male transmission efficiency; NA, not applicable.

Table S2. Primers used in this study.

MGT6ID-F	CAAACAGAAGTGCGAGTATGGTG
MGT6ID-R	CTACAAACGCAGCCGAACCTAT
CMGT6-F	AAAAGATAACTTTTTCAGAAACCTAACA
CMGT6-R	TTTGAGAGGACGACGTACAAACTAC
CMGT6ID-R	AGAGGCGAGAAAGCAGAAGAG
1300P2	GCGATTAAGTTGGGTAACGC
rtMGT6-F	CAATACTGGCGGGTACAGCA
rtMGT6-R	GAGGCTATGGTCAGCGTCAG
qMGT6-F	CAATACTGGCGGGTACAGCA
qMGT6-R	GAGGCTATGGTCAGCGTCAG
MGT5ID-F	AAATCAGAGTAGGGCGGGTTG
MGT5ID-R	GACAAGGCTAATGTAACCCCG
Tub-F	AAGGCTTTCCTTCATTGGTACA
Tub-R	CTCTCCGGCTGTAGCATCTT
MGT6-Cas9-F	GAGTCGAAGTAGTGATTGCAGCATGAATCGGTAGGGGGTTTTAGAGCTAGAAATA
MGT6-Cas9-R	TATTTCTAGCTCTAAAACCCCCTACCGATTCATGCTGCAATCACTACTTCGACTC
SALK LB1.3	ATTTTGCCGATTTCGGAAC
inMGT6-F	ATACTGGCGGGTACAGCATCAAACA
inMGT6-R	TGTATTGCTCGTCACCATACTCGCA
