# Supplementary material for: Slowing Development Facilitates Arabidopsis mgt Mutants to Accumulate Enough Magnesium for Pollen Formation and Fertility Restoration
Source: Front Plant Sci. 2021 Jan 20;11:621338. doi: 10.3389/fpls.2020.621338 (PMC7854698; doi:10.3389/fpls.2020.621338)
Supplement: Supplementary file 2 [file Data_Sheet_2.pdf]

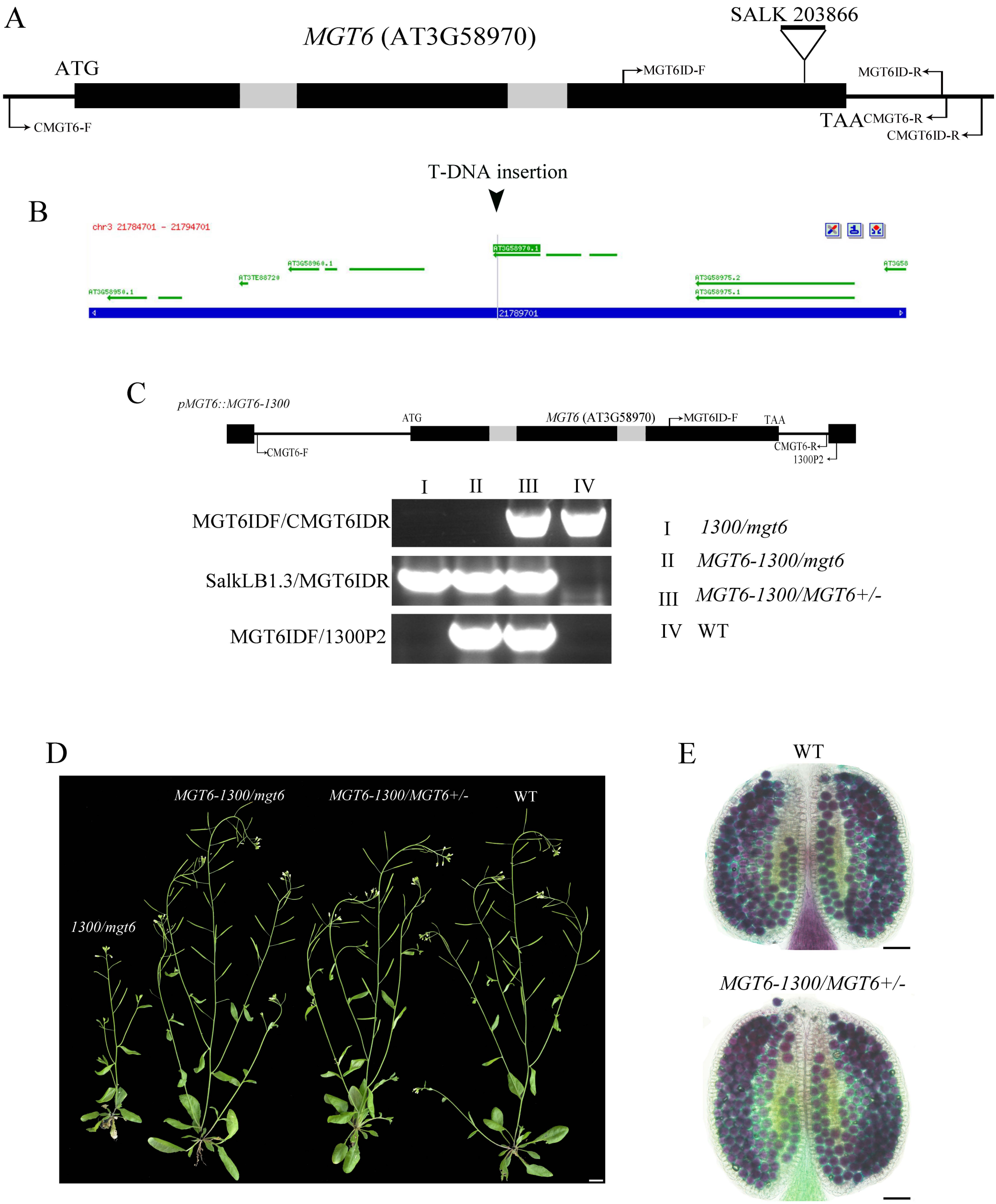

Supplemental Figure S1. T-DNA insertion site and transgenic *MGT6* complement.

(A) *MGT6* (At3g58970) contained three exons (black box). The T-DNA insertion site is indicated, and PCR amplification was performed using the primers MGT6ID-F and MGT6ID-R. **(B)** Genomic sequencing of the *mgt6* mutant revealed one T-DNA insertion in the third exon of *MGT6*. **(C)** The DNA fragment of the *MGT6* complementation mutant was cloned by primers CMGT6-F and CMGT6-R. The transgenic complementation plants were identified by PCR. **(D)** Phenotype of *MGT6* complementation plants. *MGT6* could fully complement the phenotype of *mgt6* and *MGT6+/-*. Bars, 1.5 cm. **(E)** Alexander staining of the anthers of WT and *MGT6 MGT6+/-* complementation plants. Bars, 1 mm.

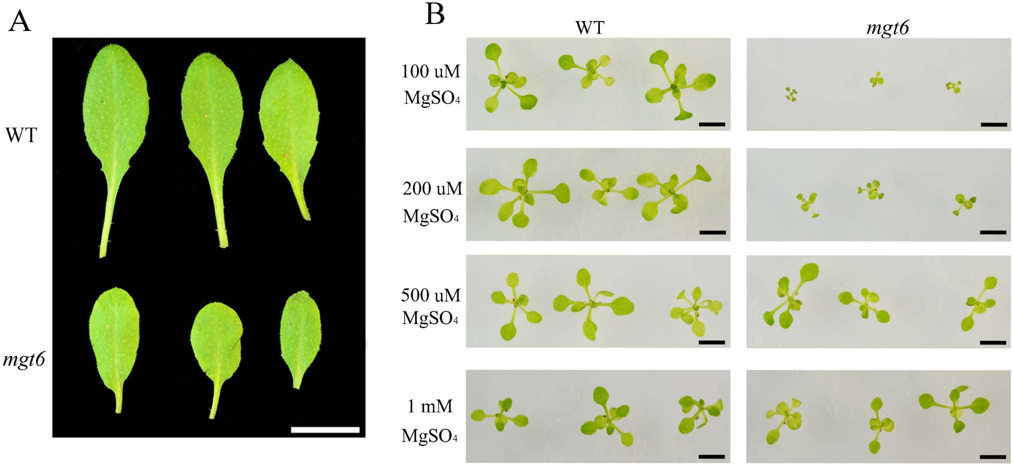

Supplemental Figure S2. Phenotype of *mgt6* under different Mg conditions.

(A) Size of leaves of WT and *mgt6* under 100 µm Mg conditions. **(B)** Young seedlings of WT and *mgt6* under different Mg conditions. The developmental defects of *mgt6* were rescued under 1 mM Mg conditions. Bars, 1 cm.

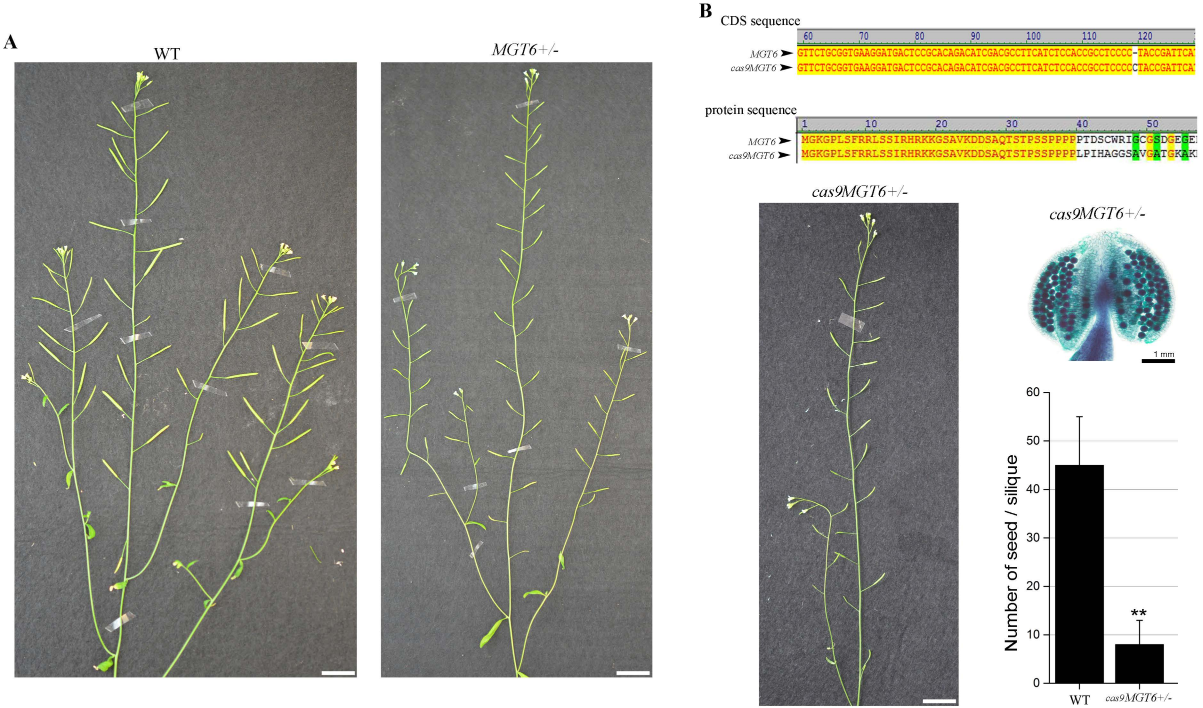

Supplemental Figure S3. Phenotype of *MGT6+/-* and *cas9MGT6+/-* mutants.

(A) WT and T-DNA insertion mutants of *MGT6+/-* plants were grown in soil supplied with 100 µM Mg. The *MGT6+/-* plants have short siliques, indicating reduced fertility. Bars, 1.5 cm. **(B)** CRISPR/Cas9 was used to generate a heterozygous mutant of *MGT6* (*cas9MGT6+/-*) that has reduced fertility. PCR-based sequencing indicated a frame-shift mutation in the codon region of *MGT6*. The *cas9MGT6+/-* plants show short siliques with normal vegetative development. Alexander staining indicated that most pollens were abortive in the anthers of the *cas9MGT6+/-* mutant. The numbers of seeds in each silique indicating the fertility of *cas9MGT6+/-* were severely reduced. Bars, 1.5 cm. The means are shown with ± SDs of two biological repeats, n > 10. A two-sample *t*-test was used to evaluate statistical significance compared with the WT (**P < 0.01).

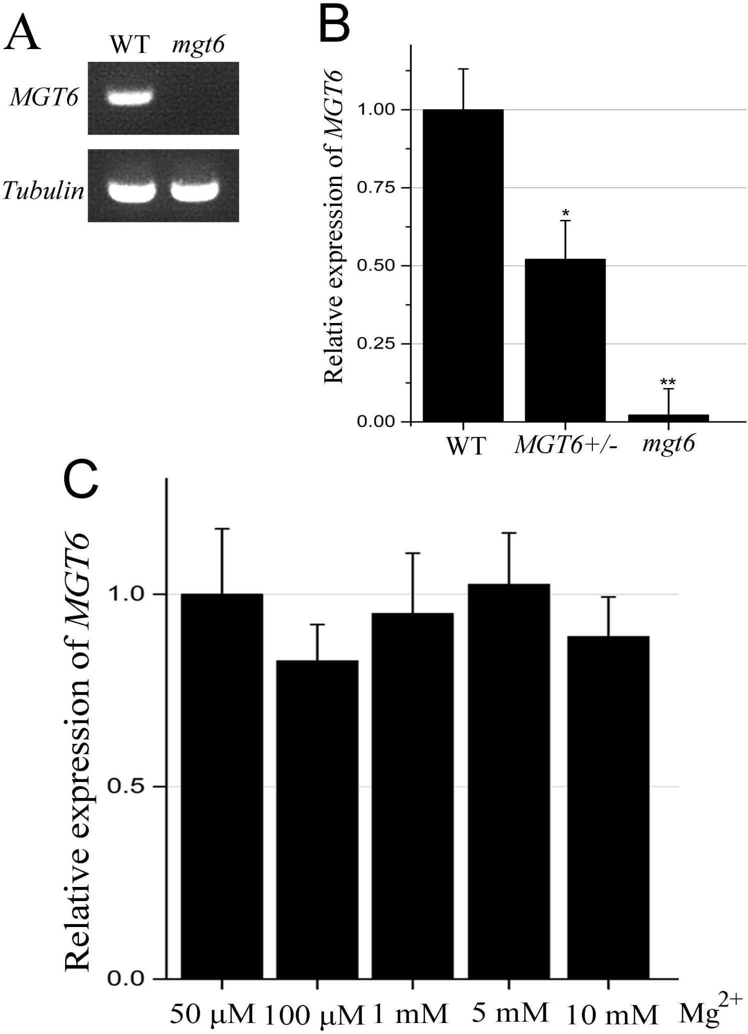

Supplemental Figure S4. Expression of *MGT6* in *mgt6*, *MGT6*+/- and WT under different Mg conditions.

(A) The *MGT6* transcript was not be detected in the *mgt6* inflorescences. (B) Expression of *MGT6* in the WT, *MGT6*+/- and *mgt6* mutant inflorescences as measured by quantitative RT-PCR. The expression is normalized to that of Tubulin and is presented relative to *MGT6* expression in the WT. The data are presented as the means ± SDs of three biological replicates. A two-sample t-test was used to evaluate statistical significance compared with the WT (*P < 0.05, **P < 0.01). (C) qRT-PCR-based analysis of *MGT6* expression in WT flower buds under different Mg conditions. The WT plants were grown in the hydroponic cultivation system under long days in the presence of 50 μM, 100 μM, 1 mM, 5 mM and 10 mM magnesium sulfate. The expression level is normalized to that of Tubulin and compared with that of the WT at 50 μM. The error bars indicate SDs and were calculated from three biological replicates.

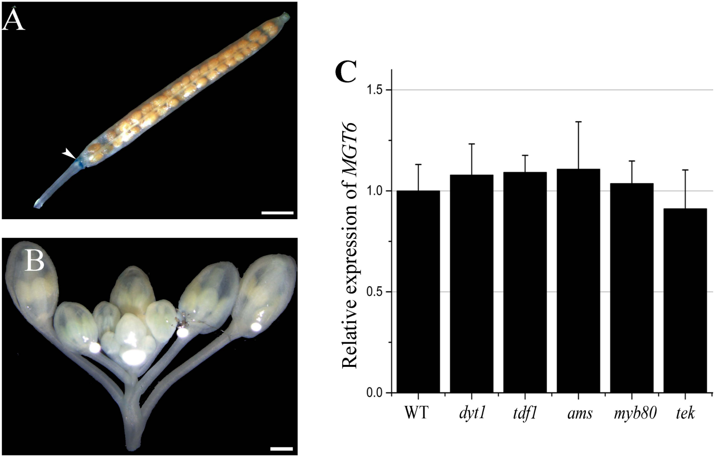

Supplemental Figure S5. GUS staining of *promoterMGT6::GUS* and WT siliques, and the expression of *MGT6* in different tapetum defective mutants.

(A) GUS signal was observed in siliques of plants transformed with the *promoterMGT6::GUS* construct. The white arrowhead indicates GUS staining. (B) No observable GUS signal was detected in the WT inflorescences. Bars, 1 mm. (C) Expression of *MGT6* in the WT, *dyt1*, *tdf1*, *ams*, *myb80* and *tek* mutants inflorescences as measured by quantitative RT-PCR. The expression is normalized to that of Tubulin and is presented relative to *MGT6* expression in the WT. The data are presented as the means ± SDs of three biological replicates.

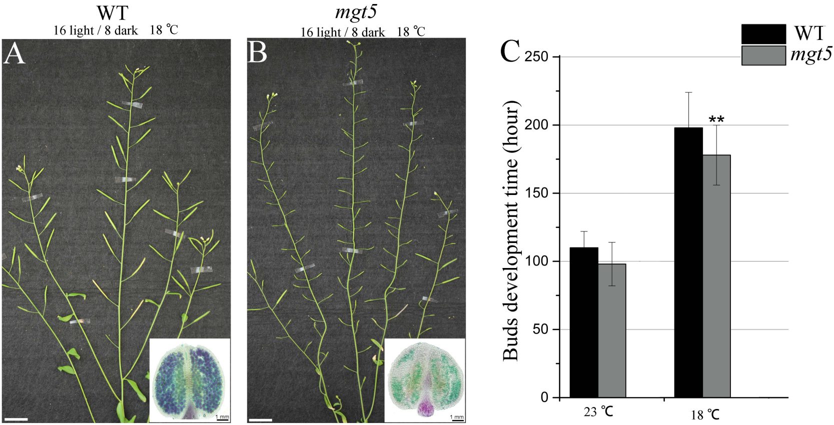

Supplemental Figure S6. Phenotypes of WT and *mgt5* under low-temperature conditions. (A-B) WT and *mgt5* were grown under 18 °C conditions. The photoperiod was set to 16/8 hours (light/dark). Bars, 1.5 cm. (C) Quantitative analyses of flower bud development time for WT and *mgt5* under 23 °C and 18 °C conditions. The means are shown as ± SDs of three biological repeats, n > 30. A two-sample *t*-test was used to evaluate statistical significance compared with the *mgt5* under 23°C (**P < 0.01).

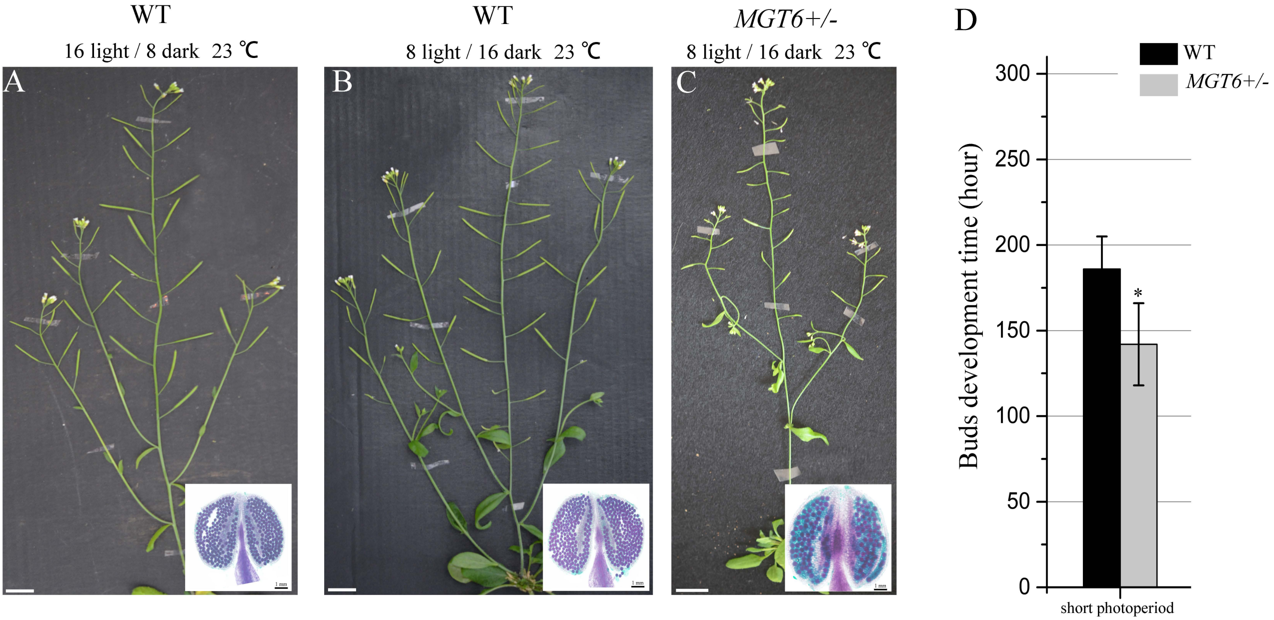

Supplemental Figure S7. Phenotypes of WT and *MGT6+/-* under short-photoperiod conditions.

(A-C) WT and *MGT6+/-* were grown under long-photoperiod and short-photoperiod conditions. The photoperiod was set to 16/8 h (light/dark) or 8/16 h (light/dark). Bars, 1.5 cm. **(D)** Quantitative analyses of flower bud development time for WT and *MGT6+/-* under short-photoperiod condition. The means are shown as ± SDs of three biological repeats, n > 10. A two-sample *t*-test was used to evaluate statistical significance compared with the WT (*P < 0.05).

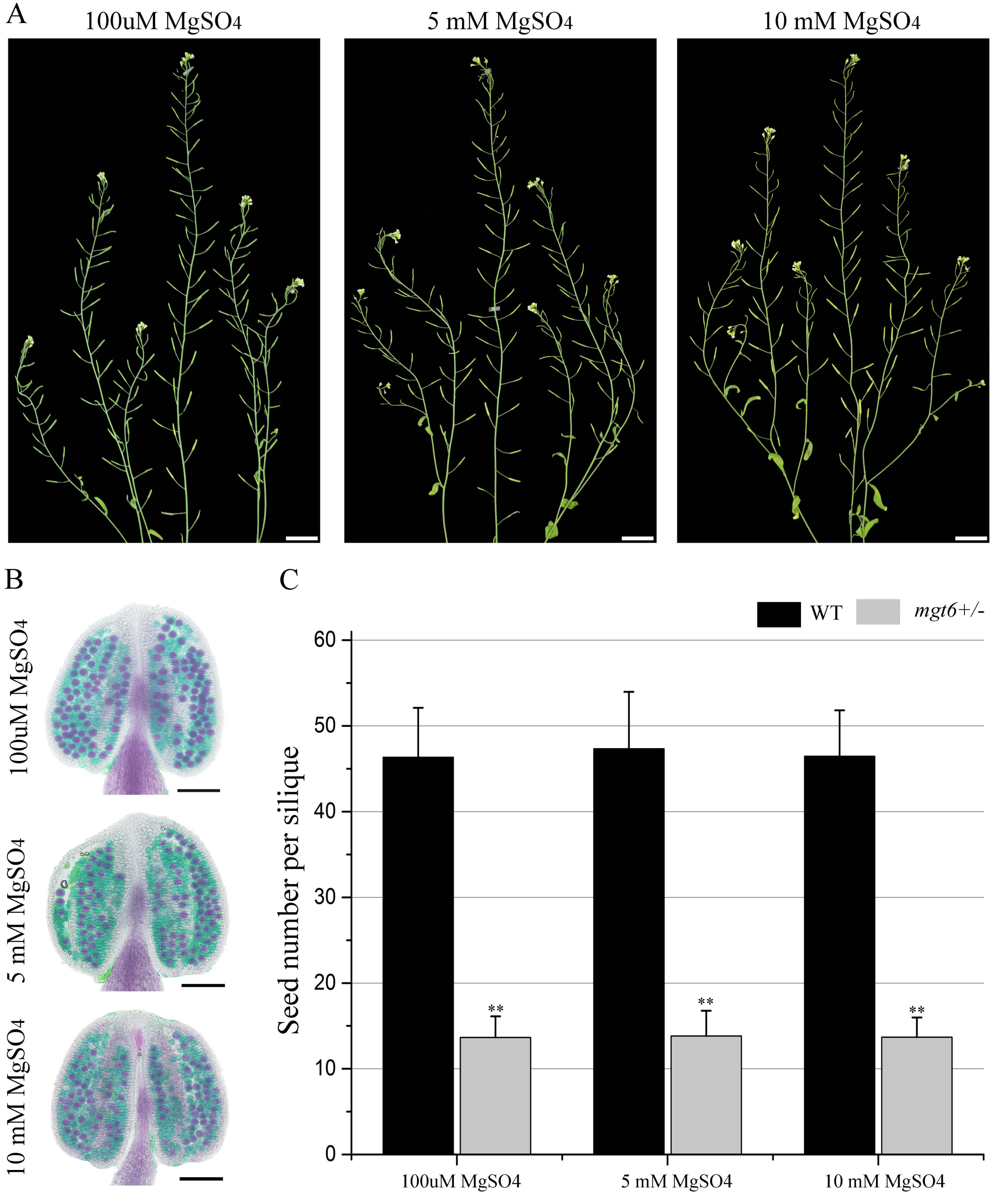

Supplemental Figure S8. Phenotype of *MGT6+/-* under different Mg conditions.

(A) *MGT6+/-* plants grown in the hydroponic cultivation system under normal photoperiod in the presence of 100 µM, 5 mM, and 10 mM magnesium sulfate (Mg). Bars, 1.5 cm. **(B)** Alexander staining of the anthers from *MGT6+/-* plants grown in the presence of different Mg concentrations. Bars, 1 mm. **(C)** The number of seeds in each filled silique from WT and *MGT6+/-* plants grown in the presence of different Mg conditions. The means are shown with ± SD for three biological repeats, n > 10. A two-sample *t*-test was used to evaluate statistical significance compared with the WT (**P < 0.01).
